# Evaluation of expert skills in refinery patrol inspection: visual attention and head positioning behavior

**DOI:** 10.1016/j.heliyon.2022.e12117

**Published:** 2022-12-07

**Authors:** Ryota Takamido, Satoya Kurihara, Yasushi Umeda, Hajime Asama, Seiji Kasahara, Yuichi Tanaka, Seigo Fukumoto, Toshiya Kato, Masahiro Korenaga, Misaki Hoshi, Jun Ota

**Affiliations:** a Research into Artifacts, Center for Engineering (RACE), School of Engineering, The University of Tokyo, Japan; b Department of Precision Engineering, Faculty of Engineering, The University of Tokyo, Japan; c Department of Precision Engineering, School of Engineering, The University of Tokyo, Japan; d Engineering & Capital Planning Department, ENEOS Corporation, Japan; e IT Planning & Development Department, ENEOS Corporation, Japan

**Keywords:** Visual inspection, Eye tracking, Virtual reality

## Abstract

We aimed to clarify expert skills in refinery patrol inspection using data collected through a virtual reality experimental system. As body positioning and postural changes are relevant factors during refinery patrol inspection tasks, we measured and analyzed both visual attention and head positioning behavior among experts and “knowledgeable novices” who were engaged in the engineering of the refinery but had less inspection experience. The participants performed a simulated inspection task, and the results showed that 1) expert inspectors could find more defects compared to knowledgeable novices, 2) visual attention behavior was similar between knowledgeable novices and experts, and 3) experts tended to position their heads at various heights and further from the inspection target to obtain visual information more effectively from the target compared to knowledgeable novices. This study presented the differences in head positioning behavior between expert and novice inspectors for the first time. These results suggest that to evaluate the skills used in inspecting relatively larger targets, both visual attention and head positioning behavior of the inspectors must be measured.

## 1 Introduction

Defect detection is a fundamental human cognitive skill which is used to identify potential defects or abnormalities in the environment. This skill can be applied to various human activities and occupations, such as the identification of defective line products (Rebsamen et al., 2010), or illegal materials during baggage checking (Sterchi et al., 2019). Since it is important to increase anomalies’ detection rates for improving the quality of these human activities, many previous studies have attempted to clarify the differences between expert inspectors and novices in various inspection tasks. For this purpose, the “expert-novice experiment paradigm” has been used, which compares inspection behavior in the same controlled environment and experimental task (Underwood et al., 2002; Hosking et al., 2010; Sharif et al., 2012; Keskin et al., 2020; Papesh et al., 2021).

Most of these studies dealt with visual inspections; therefore, they measured and compared the visual attention behavior of experts and novices using an eye tracking device (Holmqvist et al., 2011). The results of these studies revealed significant differences in visual attention behaviors, such as the frequency of changing the gaze target (Brunyé et al., 2019), attention allocation toward each area of interest (AOI) in the environment (Koh et al., 2011), and attention shift patterns (Law et al., 2004; Dzeng et al., 2016). Additionally, some training systems that reflect these insights have also been suggested (Sadasivan et al., 2005; Litchfield et al., 2010).

However, although visual attention is a critical factor responsible for differences in defect detection rates, measurements of this factor alone may not be sufficient for understanding expert skills in certain specific visual inspection tasks which involve larger inspection targets and require inspectors to repeatedly change their head positions. A typical example is refinery patrol inspection.

**Figure 1:**
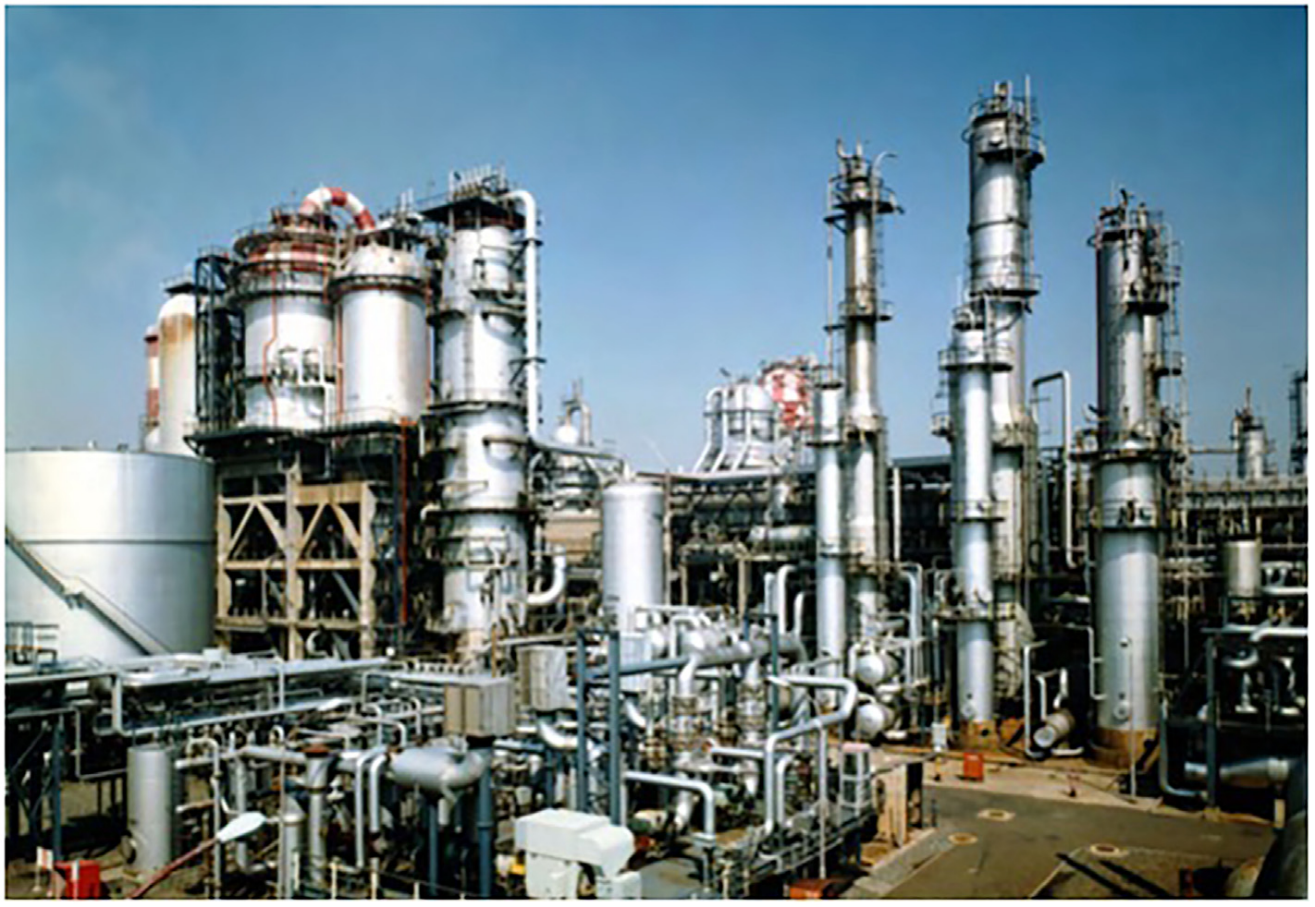
A part of the refinery. In the daily patrol inspection task, inspectors walk through these refinery areas, inspect each item of equipment, and report the defects they find (https://www.eneos.co.jp/company/about/branch/kawasaki/refinery/).

In a refinery, a daily patrol inspection is conducted by human inspectors to prevent the occurrence of serious accidents (Figure 1). These inspectors walk through the vast refinery area screening for potential hazardous defects, such as leakages or corrosions. Because of the large size of the inspection area and large number of target equipment items, it is difficult for inspectors to fit all targets into their field of view simultaneously. Hence, to inspect all targets, they need to change their head position repeatedly by performing actions that include walking, knees bending, and standing on tiptoes; variations in head position can affect the visual information obtained from gazing at the target and the detection ratio.

Therefore, it can be assumed that appropriate head positioning by performing certain actions, in addition to gazing at the appropriate target, is also a critical skill. However, to the best of our knowledge, no study has clarified the expert skills involved in the visual inspection task from specific viewpoints owing to limitations in experimental controlled experimental settings such as requirements for maintaining the participant’s head in a fixed position or a relatively smaller inspection target that does not require any actions. This is also the case in recent state-of-the-art experimental studies, e.g., Papesh et al. (2021) or Aust et al. (2022).

From an educational and practical viewpoint, it would be worthwhile to clarify the relationship between those two factors and the knowledge of the inspectors. This is because, while expert skills are currently transferred to novices through written documents such as manuals and protocols, motor skills such as positioning the head appropriately through certain actions, are generally considered to be “tacit skills” that are difficult to explain verbally (Mohajan, 2016). Conversely, recent studies have suggested that prior knowledge of the inspection target, e.g., high-risk sites, influences the visual attention strategy, even if experience with actual inspection work is lacking (Aust et al., 2021a). Therefore, knowledge of the procedures and manuals may improve cognitive skills such as visual attention in novices but may not improve motor skills, such as setting the head in the proper position. Clarifying which expert skills can and cannot be learned from written/verbal instructions is important for developing educational programs and creating effective procedures for novices.

Therefore, we measured and analyzed both the head and gaze positions during a simulated refinery inspection task by using a virtual reality (VR) experimental system; this allows the participant’s head to move freely in the environment. In addition, to obtain further insights into the relationship between those two factors and the knowledge of the refinery, we recruited and compared expert inspectors and “knowledgeable novices” who engaged in the development and engineering of the refinery and thoroughly understood the inspection materials but had less inspection experience.

Specifically, we tried to test four hypotheses. First, whether expert refinery patrol inspectors can find a larger number of defects in simulated experimental tasks, similar to experts in other inspection tasks (Hypothesis 1) as reported by Brunyé et al. (2014). Second, regarding visual attention behavior, whether novices with sufficient knowledge of the refinery show similar attention distributions, such as longer gazing durations in higher-risk components (Hypothesis 2).

**Figure 2:**
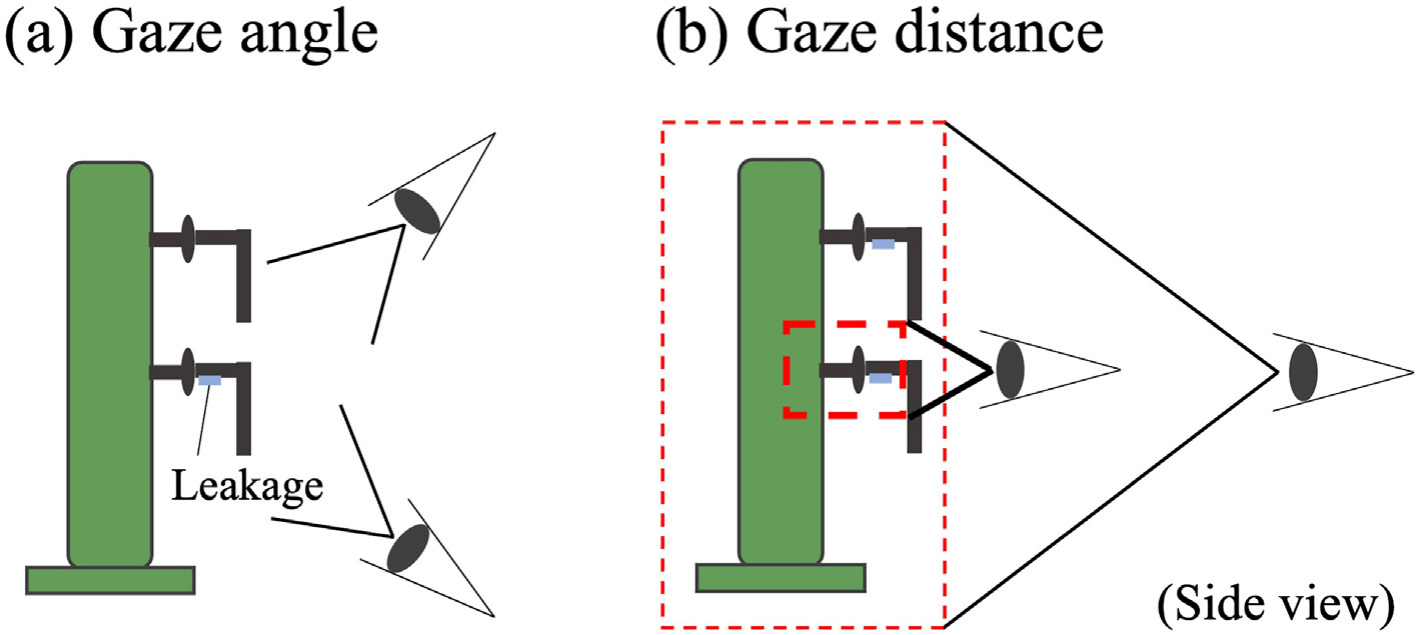
Head positioning parameter’s effects on the defect detection ratio in refinery inspection tasks. (a) Gaze distance and (b) head height.

In addition, regarding head positioning behavior, we identified two parameters that critically influence the defect detection rates: gaze distance from the target and head height. Gaze distance affects the resolution and target numbers viewing in the field of view. For example, to observe the target at high resolution, inspectors must move their heads close to it, but if the distance is too close, there is an increased risk of missing defects elsewhere at the same time (Figure 2 (a)). In general, novices need more information to make perceptual judgments than experts (Feltovich et al., 2006). Hence, we investigated whether novices tend to get closer to the target to get more detailed information to make perceptual judgments (Hypothesis 3).

Finally, head height is also a critical factor for defect detection in refinery inspection tasks. In the refinery, there are components at various heights (Figure 1); hence, inspectors must change their head height to get effective visual information. For example, in the case of a liquid leakage inspection, a main task involving visual inspection in a refinery, the detection rate of leakages is greater if an inspector looks at target equipment such as a pipe from the bottom angle by performing actions such as bending at the knee (Figure 2 (b)). A previous study showed that an improper viewing angle leads to significantly decreased detection rates (Aust et al., 2022). These head height adjustment behaviors are also considered tacit motor skills learned from genuine inspection experience. Hence, we tested if the more frequent head height changes for each inspection target among experts elicit large variances and if novices who tend to look at each target from the same head height (and pose) would elicit smaller variances (Hypothesis 4).

In this context, the purpose of our study was to clarify the differences between experts and knowledgeable novices through the verification of the above four hypotheses in refinery patrol inspections, taken as a typical example of a specific inspection task that requires inspectors to adjust both visual attention and head positioning. Although this study was conducted with a small sample size owing to the specificity of the participants, we believe that this study has potential value as a pilot to clarify the expert skills involved in visual inspection tasks from these novel aspects.

## 2 Materials and methods

### 2.1 Participants

Six male expert refinery patrol inspectors aged 23–34 years (mean years of inspection experience, 4.8 ± 1.0) and three novices aged 29–60 years participated in this study (two men and one woman). All participants had normal or corrected-normal vision. This study was approved by the ethics committee of the School of Engineering of the University of Tokyo. All study procedures conformed with the principles of the Declaration of Helsinki. Written informed consent was obtained from all participants, who were free to withdraw from the study at any time.

The novice participants were involved in the engineering and technical support of the refinery and had extensive knowledge of the locations where defects frequently occur and the technical issues written in manuals and protocols; however, they had less experience than experts in performing an actual inspection. Therefore, we speculated that by comparing these knowledgeable novices and expert inspectors, we could also clarify the relationships between knowledge and both visual attention and head positioning behavior. The number of participants and the dependent variables for the analysis were pre-defined based on the results of a pilot experiment, pre-investigation and analysis of a video recording of inspection behavior of expert and novice inspectors in a real refinery environment.

### 2.2 Experimental system

**Figure 3:**
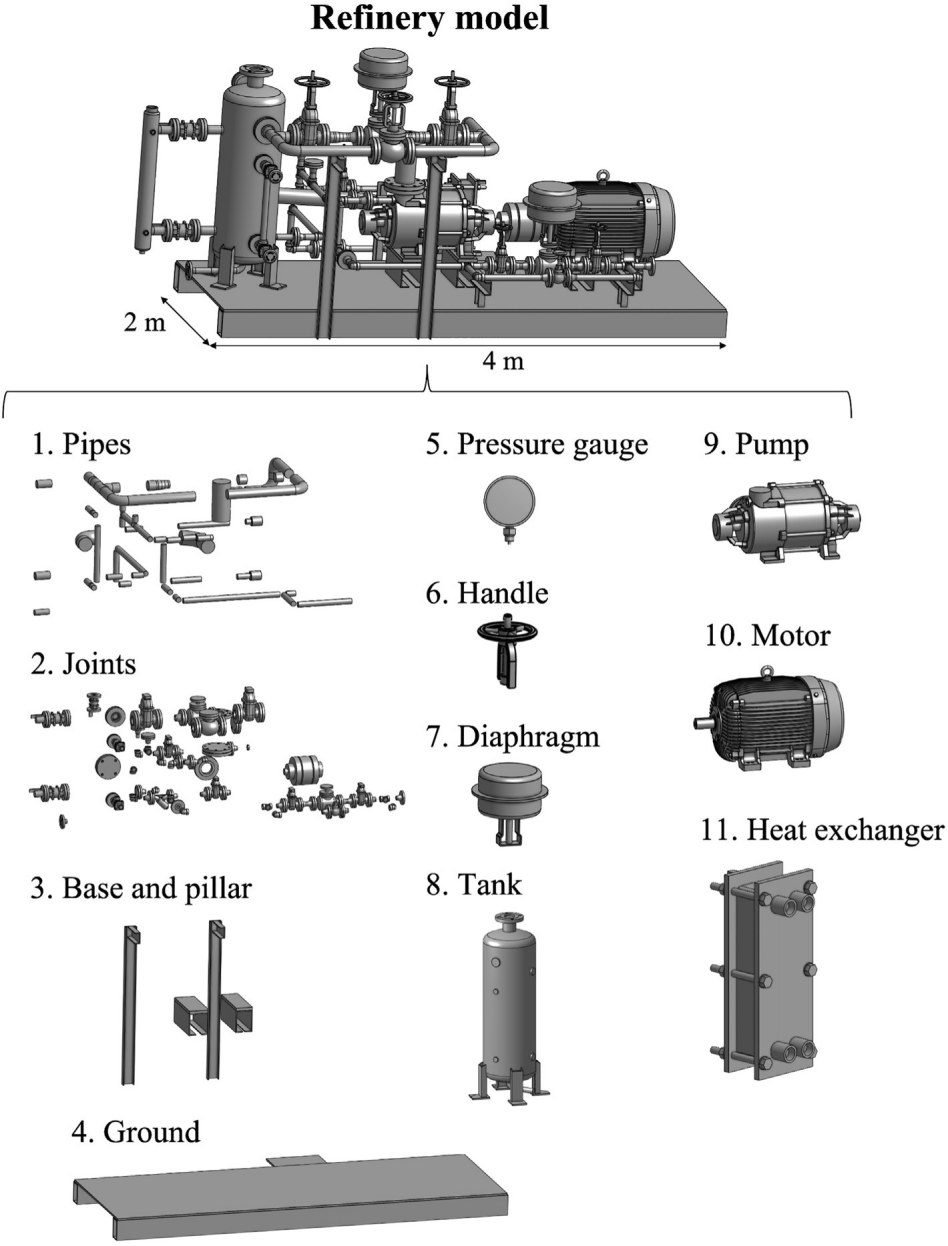
Refinery model and its components.

**Figure 4:**
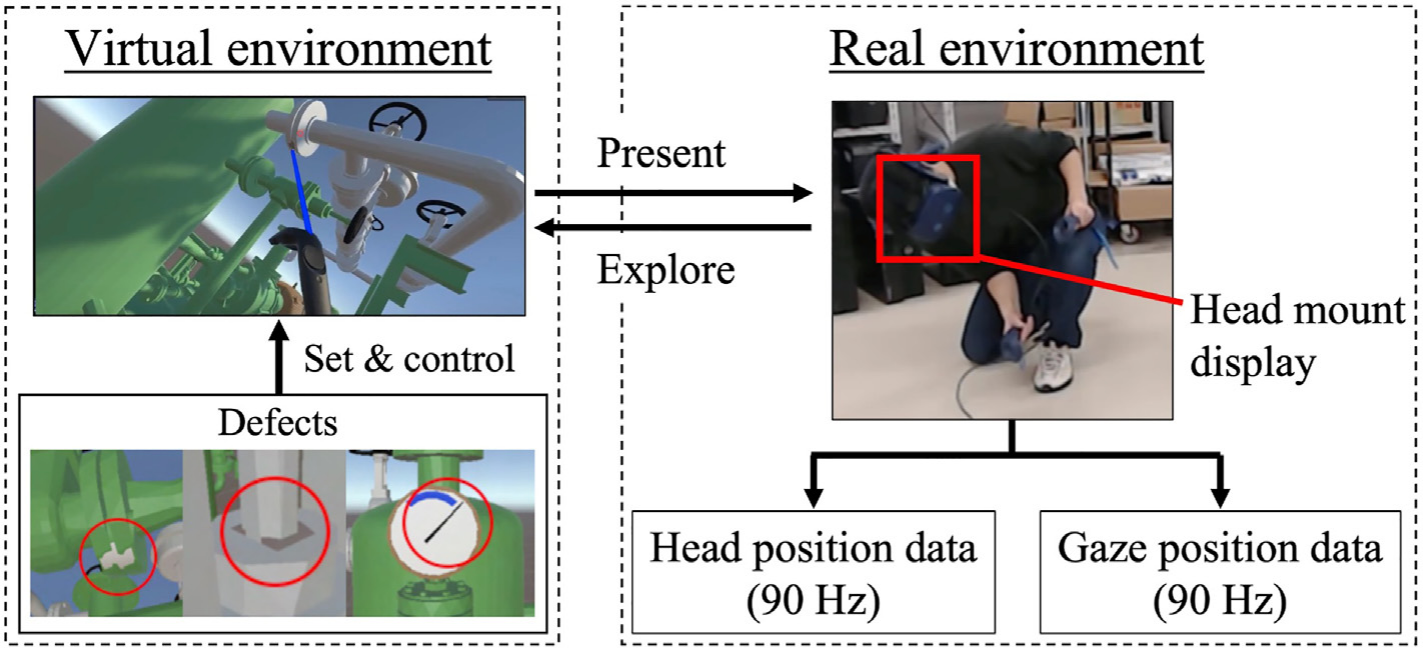
Schematic image of the experimental system.

In this study, we constructed a VR system to compare the inspection behaviors of expert inspectors and novices in the same controlled environment. Figure 3 shows the refinery model that we integrated into the virtual environment. Figure 4 shows the schematic image of the experimental system. We downloaded a part of the 3D refinery model from GrabCAD (https://grabcad.com), which includes the sites, such as joints (seals) and pipes, where defects frequently occur, and we integrated it into the virtual environment at a real scale (4 m × 2 m) using the Unity game engine (Unity Technologies, San Francisco, CA, USA). Although we only used part of the refinery model, this model contains major components such as transportation parts (e.g., pipes and joints), compressing parts (e.g., pumps), storage parts (e.g., tanks), instrumentation device parts (e.g., pressure gauges) and controlling parts (e.g., handles), as shown in Figure 3. Hence, we consider that the refinery model significantly represents the real configuration.

During the inspection of this model, the inspectors had to check a large number of pipes, joints, and other items. Additionally, we set up nine defects, assuming a liquid leakage inspection, such as leaks in joint components, including gland packings and mechanical seals, or associated abnormal values on pressure gauges. Figure 4 shows examples of these defects. The position and number of the defects are reproduced based on past defects and accidents in refineries (Shaluf et al., 2003; High Pressure Gas Safety Institute of Japan, 2018) and the inspection manuals of the refinery’s management company.

During the inspection task in this system, the model is presented to the participants through a head mount display (HMD) (HTC Vive Pro Eye HTC Corporation, New Taipei City, Taiwan), and they can move around and perform the inspection task in a similar fashion to that of the real environment. Changes in head position are directly reflected by changes in views presented through the HMD, which are measured by a system consisting of two lighthouses at 90 Hz. The HTC Vive tracking system has high spatial (several cm) and temporal precision (several tens of ms) (Niehorster et al., 2017). Additionally, the position of the participant's gaze at the presented scene was also measured by the eye tracker inside the HMD at 90 Hz. A previous study reported that the accuracy of eye tracking in HTC Vive is approximately 1.15°, and the latency is approximately 50 ms in the “head-free” condition (Sipatchin et al., 2021). Further, a first-person view video with the gaze position highlighted with a red marker is also output simultaneously with these data, which allowed us to confirm the gaze target component easily.

### 2.3 Task and procedure

By using the above experimental system, we performed the experiment to clarify expert skills in the refinery patrol inspection task, which simulated a liquid leakage inspection. The experimental task involved finding as many defects as possible in the refinery model within 2 min. When participants found defects, they reported their location by pressing a button on a handheld controller and pointing at the location with a laser beam. They performed a total of 10 experimental trials: three trials included one defect, three included two defects, and the remaining four trials were set up as a control condition with no defects. The number of defects was relatively smaller than that of previous studies (e.g., Dzeng et al., 2016); however, it is rare to find defects in an actual refinery patrol inspection. Hence, to ensure task fidelity, we set a relatively smaller number of defects for each trial. A control condition was set up to prevent the participants from predicting the number of defects. The conditions were presented in a randomized order.

First, the participants were provided with an explanation about the experiment; subsequently, they performed practice trials to familiarize themselves with the experimental system. During the practice trials, they could move around freely in the VR environment and gain an understanding of the structure and components of the model. To help participants understand how the defects are presented in the VR system, we included typical examples of defects placed in easily discoverable locations, such as at eye level; however, these defects were not included in the test trials. Participants were allowed to perform the practice trials until they acquired full understanding of the structure of the model. After completing the practice trials, they were asked to take a short break, after which they performed the test trials. They were not provided feedback regarding the location and number of defects until all test trials were completed. After completing all test trials, to verify the feeling of reality and fidelity of experts for the experimental tasks, refinery model, and defects, expert participants were asked to answer the questionnaire shown in Table 1.

**Table 1:** Questionnaire used after completing all test trials to verify the experimental settings (1, strongly disagree; 5, strongly agree).

Q1. The refinery model presented in the experiment is similar to the real one.	1・ 2 ・ 3 ・ 4 ・5
Q2. The experimental task simulates an actual inspection process well.	1・ 2 ・ 3 ・ 4 ・5
Q3. It felt more difficult to find defects in the experiment than in an actual inspection task.	1・ 2 ・ 3 ・ 4 ・ 5

### 2.4 Analyses

#### 2.4.1 Analysis 1: number of defects detected

To verify Hypothesis 1, we compared the number of defects detected in the experiment by experts and novices. We calculated the mean number of defects detected and standard deviation between the participants in the two groups; expert inspectors (*n* = 6) and novices (*n* = 3).

To calculate a valid p-value with our small sample size (total of 9 participants), we used the nonparametric pooled bootstrap t-test (Efron and Tibshirani, 1994) to verify whether the number of defects found in the experiment significantly differed between the two groups. The nonparametric pooled bootstrap t-test iterates randomly, generating a new dataset by resampling from the original dataset and calculating a t-value for the pre-defined iteration number (e.g., 1000); the p-value is defined by calculating the ratio that a t-value calculated in each iteration greater than the one calculated from the original dataset. The nonparametric pooled bootstrap t-test has higher feasibility and validity for testing a null hypothesis with a small sample size (Dwivedi et al., 2017). We pooled data of all participants, ran 1,000 nonparametric bootstrap samplings, and conducted the bootstrap t-test. These analyses were conducted with R Statistical Software (R Development Core Team, 2016; Vienna, Austria), with significance set at p < 0.05.

To confirm the validity of the results more strictly, we also calculated the effect size (Cohen’s *d*) and 95% confidence intervals (CI), considering the size of our sample (Grissom and Kim, 2005). If the CI of the effect size spans zero, the null hypotheses cannot be rejected (Coe, 2002). In other words, in our study, if the CI of the effect size did not include zero, the expertise produced positive effects. Hence, if the effect size of the expertise had been large enough to demand the above criteria, it would have been possible to detect the effect, even with data from a small sample.

#### 2.4.2 Analysis 2: allocation of visual attention

To verify Hypothesis 2, we compared the allocation of visual attention to each component in the model (Figure 3) between expert inspectors and novices during the experimental task, based on the gaze position-related data acquired from the eye tracker. Previous studies have shown that inspectors tend to pay more attention to higher-risk sites and fix their eyes on these sites for a longer duration (Koh et al., 2011). In our refinery model, components involved in transporting liquids and gases, such as joints, pipes, and handles, are considered more likely to have defects, such as leaks, than other components (Figure 3). Hence, if Hypothesis 2 were to be correct, then both experts and knowledgeable novices tend to fix their gazes on risky components for longer.

Specifically, we first defined the eye fixation time as the time during which eye velocity was below 150°/s (20 pixels/s) for at least 0.2 s continuously. For missing data within 0.1 s, a linear interpolation was performed. Subsequently, the target component at each fixation timepoint was manually identified based on the gaze position data and first-person view video. When it was difficult to identify the gaze target (e.g., gaze bordering two components), the data at that timepoint were excluded from the analysis. Next, the allocation of visual attention to the 11 components for each participant was defined by calculating the ratio of the fixation time on each component to the total fixation time. To verify the differences in the attention ratio toward each component between experts and novices, we performed a two-way analysis of variance (ANOVA) (2 groups × 11 components), and Holm-Bonferroni multiple comparisons post-hoc with the nonparametric pooled bootstrap t-test to determine the level of significance. The effect size, $\eta^{2}$, was calculated for the two-way ANOVA, and Cohen’s *d* and related CI were also calculated for the post hoc comparisons.

#### 2.4.3 Analysis 3: gaze distance and head height

**Figure 5:**
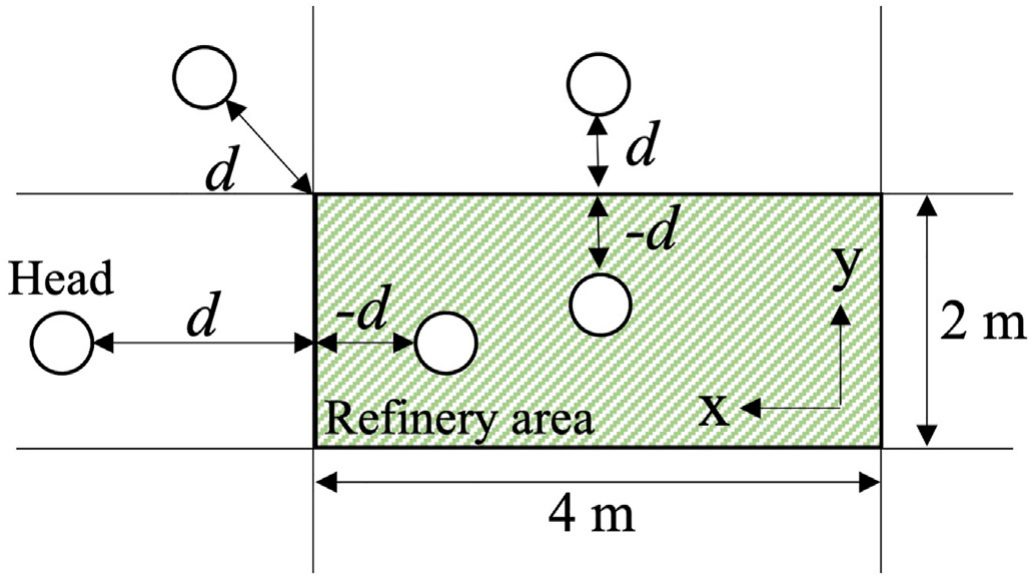
Definition of the distance from the refinery model to the inspector’s head.

To verify Hypotheses 3 and 4, we analyzed the head position data during the time in which the eyes were fixed. First, based on previous studies which also measured and analyzed head position using a motion tracking system (Okumura et al., 2012; Swarbrick et al., 2019), head position data were filtered with a cutoff frequency of 6.0 Hz using a fourth-order Butterworth filter. To verify Hypothesis 3, we defined the refinery area as 2 m × 4 m on the x-y plane, and the distance from it was measured as shown in Figure 5. Negative values were assigned when the head was positioned within this area. Subsequently, we defined three distances from the model: “close,” “middle,” and “far,” where “close” represented a distance < -0.25 m, “far” represented a distance >0.25 m, and “middle” represented a distance between these two (−0.25–0.25 m). We defined these distances based on the previously mentioned practical aspects of the inspection task (see Introduction). In other words, “close” means that the inspector gets closer to the inspection target observing details of it, whereas “far” means that the inspector moves away from the model and attempts to observe the inspection target in its entirety. The distance thresholds (−0.25, 0.25 m) were set assuming about one shoe length difference from the neutral state. During the statistical analysis, to verify the effect of expertise on gaze distance, we performed multiple nonparametric pooled bootstrap t-tests to compare the differences in the ratio of the three levels; additionally, the Bonferroni adjustment was used for multiple comparisons, and the effect size and CI were also calculated.

Additionally, to verify Hypothesis 4, we also compared head height. Specifically, the height of each participant was used as a reference (1.0), and head height data during eye fixation were converted to a ratio and recorded. We defined three levels of head height: “high,” “middle,” and “low,” where “high” represented the position at which the participant’s head was higher than 0.8, “low” the position at which it was lower than 0.5, and “middle” the position at which it was located between these two levels (0.5–0.8). These criteria were defined based on the average height of the shoulder (approximately 0.8) and hip (approximately 0.5) (Plagenhoef et al., 1983). Next, we calculated the ratio of these three levels to the total fixation time in each participant; subsequently, we calculated the average ratio of the two groups. The statistical analysis performed was the same as that of the gaze distance.

## 3 Results

The results of the questionnaire showed that the mean rating was 4.0 ± 0.0 for Q1 (reality of the refinery model), 3.6 ± 1.4 for Q2 (reality of the experimental task), and 4.7 ± 0.8 for Q3 (difficulty of the experimental task). Although the experts perceived the experimental task to be difficult, they also perceived the refinery model and simulated inspection task in the virtual environment to be rather realistic.

### 3.1 Analysis 1: number of defects detected

**Figure 6:**
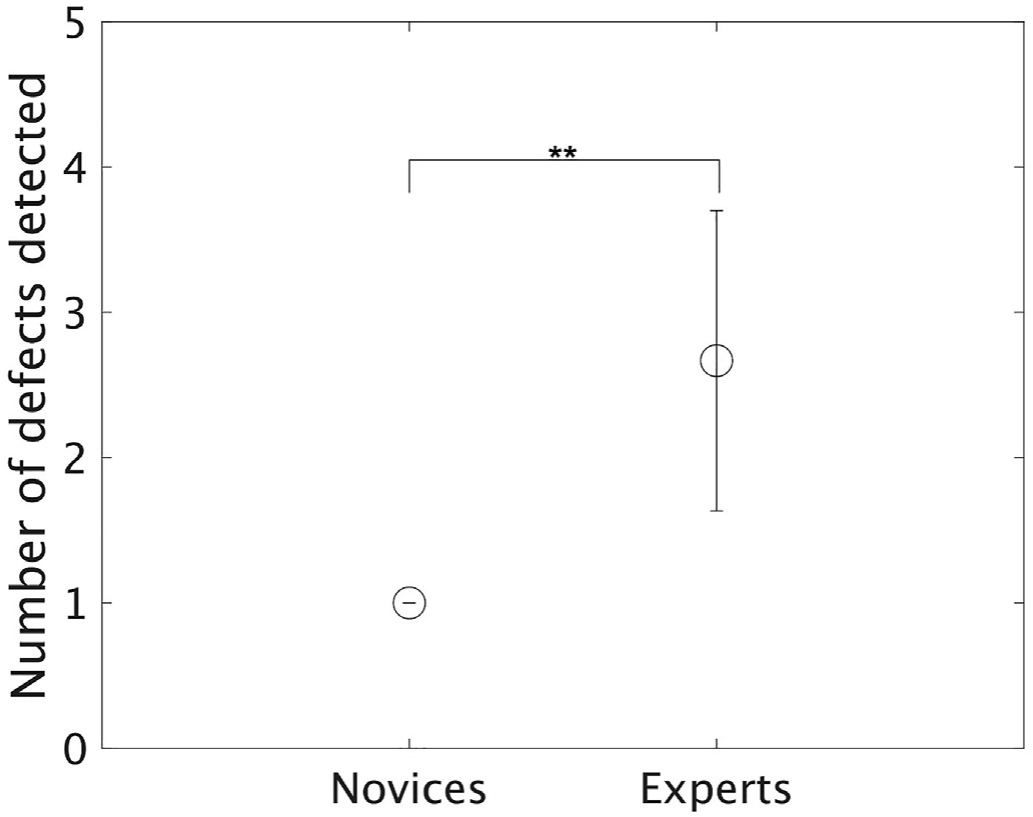
Average number of defects detected by the experts and novices (∗∗: p < .01). The error bars represent the standard deviations.

The average number of defects detected was 2.7 ± 1.0 (30 ± 11%) for the expert inspectors and 1.0 ± 0.0 (11%) for the novices (Figure 6). All novices found only one defect. The nonparametric pooled bootstrap t-test revealed that the expert inspectors found a significantly larger number of defects than novices; further, there was a large effect size, and no CI of the effect size was below zero (*p* < .01, d = 2.01, 95% CI [0.26, 3.75]). Hence, these results supported Hypothesis 1; although the effect sizes ranged from small to very large, there were positive effects of expertise on defect detection ability.

### 3.2 Analysis 2: allocation of visual attention

**Figure 7:**
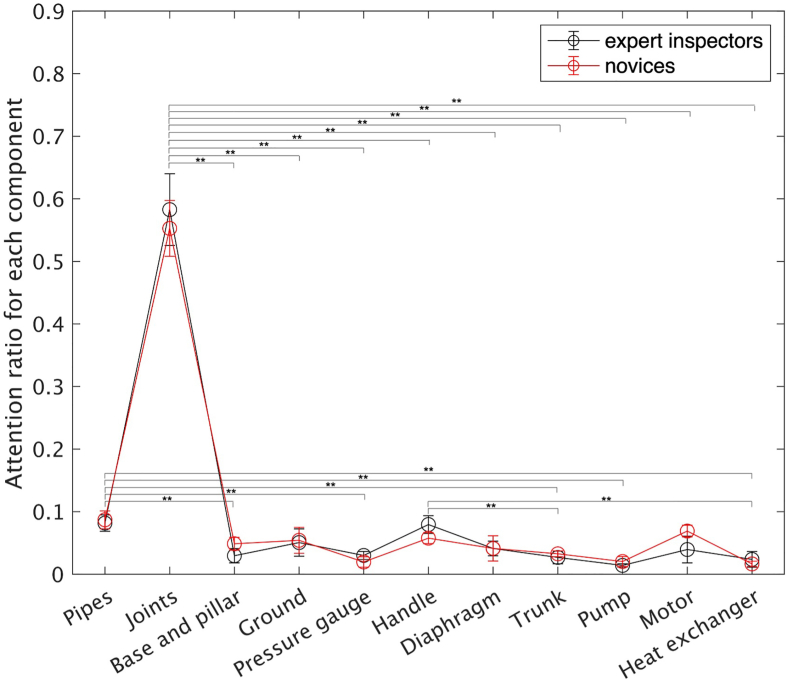
Visual attention ratio toward each component in experts and novices (∗∗: p < .01). The error bars represent the standard deviations.

Figure 7 shows the results of Analysis 2. The experts and novices had similar attention distributions. The results supported Hypothesis 2. The two-way ANOVA revealed no significance of the main effect of expertise (*p* > .05) and no interaction between the two factors (*p* > .05); however, there were significant effects of the components (*F* (*df* = 10, 77) = 322.1, *p* < .01, $\eta^{2\;}$ = 41.8). The post-hoc analysis showed that both experts and novices tended to pay more attention to the high-risk components, such as joints, than to other parts as we hypothesized (joints vs. pipes: *p* < .01, d = 8.22, 95% CI [3.69, 12.74]; joints vs. base and pillars: *p* < .01, d = 12.17, 95% CI [6.22, 20.49]; joints vs. ground: *p* < .01, d = 12.17, 95% CI [5.64, 18.69]; joints vs. pressure gauge: *p* < .01, d = 9.32, 95% CI [4.24, 14.39]; joints vs. handle: *p* < .01, d = 8.44, 95% CI [3.80, 13.07]; joints vs. diaphragm: *p* < .01, d = 9.02, 95% CI [4.09, 13.94]; joints vs. tank: *p* < .01, d = 9.27, 95% CI [4.22, 14.31]; joints vs. pump: *p* < .01, d = 9.52, 95% CI [4.34, 14.69]; joints vs. motor: *p* < .01, d = 8.77, 95% CI [3.97, 13.56]; and joints vs. heat exchanger: *p* < .01, d = 14.49, 95% CI [6.78, 22.20]). They also tended to pay more attention to the pipes than to the base and pillars (*p* < .01, d = 2.29, 95% CI [0.82, 4.91]), pressure gauge (*p* < .01, d = 3.02, 95% CI [0.91, 5.12]), tank (*p* < .01, d = 2.87, 95% CI [0.82, 4.91]), pump (*p* < .01, d = 3.65, 95% CI [1.28, 6.01]), and heat exchanger (*p* < .01, d = 3.02, 95% CI [1.05, 5.44]). Further, they paid more attention to the handle than to the tank (*p* < .01, d = 2.51, 95% CI [0.59, 4.42]) and heat exchangers (*p* < .01, d = 2.94, 95% CI [0.86, 5.01]).

### 3.3 Analysis 3: gaze distance

**Figure 8:**
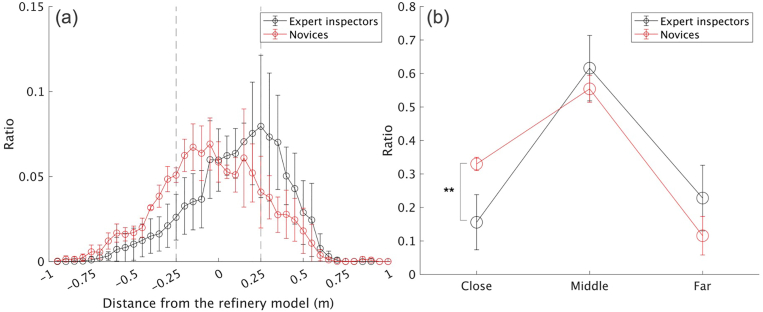
Differences in head height during the inspection task. (a) Average ratio of each 0.05 change relative to the standing height (1.0) of each participant in each group. (b) Average ratio of the three levels (∗∗: p < .01). The error bars represent the standard deviations.

**Figure 9:**
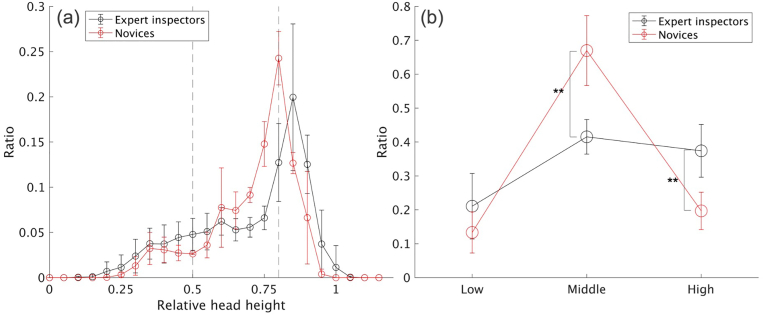
Differences in the distance from the model to the inspector’s head during the inspection task. (a) Average ratio for each 0.05 m change in distance for the refinery in each group. (b) Average ratio of the three distances (∗∗: p < .01). The error bars represent the standard deviations.

Figure 8 (a) shows the mean ratio of the distance between the head and refinery for every 5 cm during the experimental task, while Figure 8 (b) shows the mean ratio of the three head distance parameter levels; “close,” “middle,” and “far.” As we hypothesized (Hypothesis 3), expert inspectors tended to move closer to the refinery less frequently than novices (*p* < .01, d = 2.47, 95% CI [0.57, 4.36]). Figure 9 (a) shows the mean ratio of the head height for every 0.05 for the reference (each participant’s height), while Figure 9 (b) shows the mean ratio of the three head height parameter levels; “low,” “middle,” and “high.” These results also supported Hypothesis 4; expert inspectors tended to position their head at the “middle” level less frequently (*p* < .01, d = 3.64, 95% CI [1.18, 5.75]) and at the “high” level more frequently (*p* < .01, d = 2.46, 95% CI [0.56, 4.35]) than novices. Therefore, the results showed a larger expertise effect on head positioning behavior.

## 4 Discussion

In this study, we compared and clarified the differences in inspection-related behaviors between expert inspectors and novices based on the number of defects detected (Analysis 1 for Hypothesis 1), visual attention (Analysis 2 for Hypothesis 2), and head positioning (Analysis 3 for Hypothesis 3 and 4).

The results of Analysis 1 revealed that expert inspectors found a larger number of defects than novices during the experimental inspection task; however, the detection rate was relatively lower than that in previous studies (about 50–90% (Rebsamen et al., 2010; Dzeng et al., 2016; Aust et al., 2021b), even in the case of expert inspectors (approximately 19–41%). These low detection rates match the perceived task difficulty from the questionnaire. There are a few possible reasons for the decrease in the detection rate in this study. First, we limited the inspection time to 2 min; this constraint might have been too strict, making the experimental task more difficult than a real inspection. Time for inspection is a critical factor that affects the detection rate (See, 2012). Second, during an actual inspection of the refinery, inspectors can use other sensory information, such as auditory, olfactory, and tactile information, and detect defects more easily using such “multimodal” information. A recent study reported that compared with only visual information, the combination of visual and tactile feedback can improve the detection rate (Aust et al., 2021b). A lack of multimodality in the experimental system may also decrease the reality and fidelity feelings of the task for some experts. Hence, although the performance of the expert inspectors was better than that of the novices in our experimental conditions, when other sensory information is used, their detection rate and perception of reality of the virtual environment may improve, and the differences from the novices would be clearer.

Additionally, the results of Analysis 2 revealed that both the expert inspectors and novices tended to pay more attention to higher-risk components such as joints or pipes and expertise was not a significant factor involved. The novices who participated in the experiment had some prior knowledge of the refinery, which suggests that the components gazed at during the inspection task reflect such knowledge of the targets. Recently, Aust et al. (2021a) compared eye behavior during an inspection task of an aircraft’s engine blade between inspectors, engineers, and assembly operators. They showed that the attention distribution was similar between inspectors and engineers despite the engineers’ lack of inspection experience; the reason for this was prior knowledge, including that of higher-risk parts. These similarities in sites’ attention patterns between knowledgeable novices and experts match our results; hence, there is a possibility of improving the visual attention behavior of novices through teaching documents, such as manuals and written protocols.

Finally, although both experts and novices exhibited similar visual attention behaviors, the results of Analysis 3 revealed clearer differences in terms of head positioning behavior, such as adjusting the head height or distance from targets. These suggest that although both experts and novices tend to look at the same components, owing to the differences in head positioning during the visual attention behavior, the information they obtain is different; this may lead to differences in the detection rate, as shown in Analysis 1. Specifically, the novices knew “what they should have looked at” from their explicit knowledge; however, they did not know “how to look at it.” The lack of this tacit motor skill may result in them overlooking some defects. Since most previous studies only evaluated the visual attention of inspectors, these expert-novice differences in head positioning are first revealed by this study.

To summarize the results of the verification of the four hypotheses through these three analyses, our results suggest that when attempting to clarify the expert skills for an inspection task of a large target that requires inspectors to change the position and posture of their body in order to inspect all the components, it is best to evaluate the inspection behavior from both the visual attention and head positioning viewpoints.

This study has some limitations, and there is scope for future research in this context. First, although the significantly different parameters in this study had sufficiently large effect sizes that could be detected with the small sample size, generalizing the results should be carefully done since statistical significance does not necessarily imply generalizability. Hence, based on the results of this pilot study, further verification in real environment is needed in the future. Second, although most experts perceived a certain level of reality in our experimental system, our experimental settings still suffer from real-life fidelity limitations, such as lacking the previously mentioned multimodality. Further, since we only used one standard refinery model for the experiment, the generalizability of our results should be verified in further experiments. Although our experimental model was meant to represent a substantial part of a real-life environment, its number of devices and components is still limited. Finally, our main aim was to clarify how the inspection behavior of experts and novices differ, rather than why they are different. Therefore, to understand the mechanism behind these differences accurately, additional controlled experiments must be conducted based on the results of this study; these may include comparing the inspection behavior patterns between novice groups with and without specific knowledge, such as higher-risk components positions.

## 5 Conclusions

In this study, we attempted to verify four hypotheses to clarify the skills of expert refinery patrol inspectors from the viewpoints of visual attention and head positioning behavior using a VR experimental system. The results showed that expert inspectors found a greater number of defects in the experimental inspection task (Analysis 1 for Hypothesis 1); however, both experts and novices tended to pay more attention to higher-risk components (Analysis 2 for Hypothesis 2). Furthermore, significant differences in head positioning behavior, such as head height adjustments and distance from the refinery model, resulted in a variance in the visual information obtained through similar visual attention behavior; in turn, this led to differences in the detection rate between experts and novices (Analysis 3 for Hypotheses 3 and 4). Although the sample size of this study is relatively smaller than that of previous studies, our results on expertise have sufficient statistical strength, with high consistency in the results (low variance) between the expert and novice groups. Therefore, to understand the inspection skills required for large inspection targets that require inspectors to change the position and posture of their body for a thorough inspection of all components, both visual attention and head positioning behavior must be measured. This is the first study that reports the differences in head positioning behavior between expert and novice inspectors. Further, based on the results of this study, in-depth experiments and analyses are warranted.

## Declarations

### Author contribution statement

Ryota Takamido: Conceived and designed the experiments; Performed the experiments; Analyzed and interpreted the data; Wrote the paper.

Satoya Kurihara: Conceived and designed the experiments; Performed the experiments; Analyzed and interpreted the data.

Yasushi Umeda; Hajime Asama: Conceived and designed the experiments.

Seiji Kasahara; Yuichi Tanaka; Seigo Fukumoto; Masahiro Korenaga; Misaki Hoshi: Contributed reagents, materials, analysis tools or data.

Toshiya Kato: Performed the experiments; Contributed reagents, materials, analysis tools or data.

Jun Ota: Conceived and designed the experiments; Analyzed and interpreted the data.

### Funding statement

This work was partially supported by the company ENEOS Corporation.

### Data availability statement

Data included in article/supp. material/referenced in article.

### Declaration of interest’s statement

SK, YT, SF, MK, MH and TK were employed by ENEOS Corporation. YM, HA and JO have received research support from ENEOS Corporation for this work. The remaining authors declare no conflict of interest.

### Additional information

No additional information is available for this paper.

## Appendix A Supplementary data

Questionnaire(Translated from Japanese) Questionnaire(Translated from Japanese)
