# Supplementary material for: Evaluation of expert skills in refinery patrol inspection: visual attention and head positioning behavior
Source: Heliyon. 2022 Dec 7;8(12):e12117. doi: 10.1016/j.heliyon.2022.e12117 (PMC9761707; doi:10.1016/j.heliyon.2022.e12117)
Supplement: Questionnaire(Translated from Japanese) [file mmc1.docx]

Thank you very much for participating our experiment. We would like to ask a few questions about your impressions and feelings of our experiment. Note that your privacy is protected, and your answer is only used for out research.

Q1 Please answer the following questions to the extent that there is no problem.

| Name |  | Gender |  |
| --- | --- | --- | --- |
| Age |  | Years of Experience |  |

Q2 The refinery model presented in the experiment is similar to the real one (1: strongly disagree - 5: strongly agree).

1　　・　　２　　・　　３　　・　　４　　・　　５

Q3 Experimental task is a well simulated actual inspection process (1: strongly disagree - 5: strongly agree).

1　　・　　２　　・　　３　　・　　４　　・　　５

Q4 Felt more difficult to find defects in the experiment than in an actual inspection task(1: strongly disagree - 5: strongly agree).

1　　・　　２　　・　　３　　・　　４　　・　　５

This is the end of the questionnaire. Thank you for taking your time to complete this questionnaire.
